# Supplementary material for: Light-fueled transient supramolecular assemblies in water as fluorescence modulators
Source: Nat Commun. 2021 Aug 17;12:4993. doi: 10.1038/s41467-021-25299-8 (PMC8371092; doi:10.1038/s41467-021-25299-8)
Supplement: Supplementary file 1 — Supplementary Information [file 41467_2021_25299_MOESM1_ESM.pdf]

Supplementary Information

Light-Fueled Transient Supramolecular Assemblies in Water as Fluorescence Modulators

Xu-Man Chen,[a] Xiao-Fang Hou,[b] Hari Krishna Bisoyi,[c] Wei-Jie Feng,[a] Qin Cao,[a] Shuai Huang,[a] Hong Yang,*[a] Dongzhong Chen*[b] & Quan Li*[a, c]

[a]Institute of Advanced Materials, School of Chemistry and Chemical Engineering, and Jiangsu Province Hi-Tech Key Laboratory for Bio-medical Research, Southeast University, Nanjing 211189, China; [b]Key Lab of High Performance Polymer Materials and Technology of MOE, School of Chemistry and Chemical Engineering, Nanjing University, Nanjing 210023, China; [c]Advanced Materials and Liquid Crystal Institute and Chemical Physics Interdisciplinary Program, Kent State University, Kent, OH 44242, USA

Table of contents

A. Methods
B. Reversible photo-isomerization between SMEH and ASP
C. Determination of the preferable assembly concentration of ASP-CS by optical transmittance
D. Formation and Dissociation of dissipative SMEH-CS system
E. Characterization of reversibility and dissipative kinetics of SMEH-CS system
F. Chemical structure of fluorophores in this work
G. Demonstration of formation and dissociation process of the dissipative self-assemblies by loading C153
H. Confocal images of the dissipative self-assemblies by loading C153
I. Demonstration of time-dependent AIE and ACQ processes by loading different fluorophores
J. Cell cytotoxicity
K. ^{1}H-NMR, ^{13}C-NMR and HRMS spectra of SMEH
L. References

A. Methods

Materials

Chemical reagents were purchased from commercial resource unless noted. The deacetylation degree of chitosan is 95% and the average molecular weight of chitosan is 1526.5 g/mol, respectively.

Synthesis of SMEH. SMEH was synthesized according to published literature[1], 2,3,3-trimethylindolenine (1.65 g, 0.01 mmol) was firstly added into propane sultone (1.26 g, 0.01 mmol). The mixture was stirred at 90 °C for 4 h under N_2. The purple solid was collected by filtration, washed with cold diethyl ether, and dried in vacuo to generate 2,3,3-trimethyl-1-(3-sulfonatepropyl)-3H-indolium. Then, 2,3,3-trimethyl-1-(3-sulfonatepropyl)-3H indolium (100 mg, 0.36 mmol) and 2-hydroxybenzaldehyde (48 mg, 0.39 mmol) were added into anhydrous ethanol (2 mL). The mixture was allowed to reflux overnight. The orange solid SMEH was obtained by filtration. The structure was determined by ^{1}H-NMR, ^{13}C-NMR and HRMS. ^{1}H NMR (400 MHz, DMSO-d_6): δ 11.03 (s, 1H), 8.60 (d, J = 16.4 Hz, 1H), 8.28 (d, J = 6.4 Hz, 1H), 8.02 (d, J = 6.4 Hz, 1H), 7.92 – 7.82 (m, 2H), 7.67 – 7.58 (m, 2H), 7.48 (t, J = 6.9 Hz, 1H), 7.04 (d, J = 8.4 Hz, 1H), 6.99 (t, J = 7.6 Hz, 1H), 4.87 – 4.74 (t, J = 6.9 Hz, 2H), 2.65 (t, J = 6.5 Hz, 2H), 2.19 (q, J = 6.7 Hz, 2H), 1.77 (s, 6H). ^{13}C NMR (101 MHz, DMSO-d_6): δ 182.24, 159.49, 149.16, 143.96, 141.40, 136.21, 130.25, 129.62, 129.59, 123.45, 121.82, 120.53, 117.09, 115.56, 111.93, 52.38, 47.80, 46.01, 26.91, 25.07. HRMS (m/z): [M − H]$^-$ calcd. for $C_{21}H_{22}NO_4S^-$, 384.12750; found, 384.12730.

The preparation of ASP-CS transient self-assemblies induced by light. SMEH (0.15 mM) and CS (40 μg/mL) aqueous solution was firstly prepared at 25 ºC. Then the solution was exposed under 420 nm light (15 mW/cm^2 unless mentioned) for ~50 s for light-induced assembly, ASP-CS, for further measurements.

NMR spectroscopy. ^{1}H and ^{13}C NMR spectra were recorded on a Bruker 400 MHz spectrometer.

ESI-MS spectroscopy. Electrospray ionization mass spectra (ESI-MS) were measured by Agilent 1260-6224.

UV-Vis spectroscopy. UV-Vis spectra and the optical transmittance were recorded in a quartz cell (light path 10 mm) on a Shimadzu UV-2700 spectrophotometer equipped with a temperature controller.

Fluorescence spectroscopy. Steady-state fluorescence spectra were recorded in a conventional quartz cell (light path 10 mm) on a Hitachi F-4700 equipped with a temperature controller.

Transmission electron microscopy. High-resolution transmission electron microscopy (TEM) images were acquired using a Tecnai 20 high-resolution transmission electron microscope operating at an accelerating voltage of 200 keV. The sample for high-resolution TEM measurements was prepared by dropping the solution onto a copper grid. The grid was then air-dried under 420 nm irradiation for light-induced ASP-CS self-assembly, and in the dark for SMEH-CS dissociated state at room temperature.

Scanning electron microscopy. Scanning electron microscopy (SEM) images were obtained using a Hitachi S-3500N scanning electron microscope. The sample for high-resolution SEM measurements was prepared by dropping the solution onto a silicon

wafer. The wafer was then air-dried under 420 nm irradiation for light-induced ASP-CS self-assembly, and in the dark for SMEH-CS dissociated state at room temperature.

Dynamic light scattering spectroscopy. Solution samples were examined on a laser light scattering spectrometer (BI-200SM) equipped with a digital correlator (TurboCorr) at 636 nm at a scattering angle of 90°. The hydrodynamic diameter (Dh) was determined by dynamic light scattering experiments at 15 °C.

Confocal laser scanning microscopy. The images of the formation and dissociation of the ASP-CS dissipative assembly after loading C153 and its dynamic cell imaging with SRB were all obtained from Olympus FV3000 confocal laser scanning microscope.

Cytotoxicity experiments. Human hepatocellular cancer cells (HepG2) were incubated in RPMI-1640 medium. The medium was supplemented with 10% fetal bovine serum. HepG2 cells were seeded in 96-well plates (5×10^4 cell mL^{-1}, 0.1 mL per well) for 24 h at 37ºC in 5% CO_2. Then the cells were incubated with ASP-CS-SRB assemblies at corresponding concentrations for 4 h. To keep ASP-CS-SRB transient assemblies in HepG2 cells, the examples were under 420 nm irradiation (~2mW/cm^2). The relative cellular viability was determined by the MTT assay.

Dynamic HepG2 cell imaging of ASP-CS-SRB transient assemblies. HepG2 cells were seeded in 6-well plates (5×10^4 cell mL^{-1}, 2 mL per well) for 24 h at 37ºC in 5% CO_2. The cells were incubated with the ASP-CS-SRB solution for 4 h. To keep ASP-CS-SRB transient assemblies in HepG2 cells, the examples were under 420 nm irradiation (~2mW/cm^2). Then the medium was removed, and the cells were washed with phosphate buffer solution for three times. Right after that, the cells were subjected to observation by a confocal laser scanning microscope for laser scanning confocal images. Every 2 min a confocal image was taken to observe the release of fluorescent SRB through the dissociation of ASP-CS transient assemblies.

Determination of the percentage of assembled ASP in ASP-CS co-assemblies: We have investigated the percentage of assembled ASP in the solution by dialyzing the ASP-CS solution under 420 nm irradiation.

Firstly, we prepared SMEH-CS solution 5 mL for 420 nm irradiation ($[SMEH]_{initial}$ = 0.15 mM, [CS] = 40 μg/mL) to get ASP-CS co-assemblies. Then, the 5 mL solution was put into a dialysis tube for dialysis with 500 mL water out of the tube. During the dialysis process, the dialysis tube was under 420 nm irradiation (15 mW/cm^2) to keep the assembling state of ASP-CS co-assemblies. After that, the solution in the dialysis tube was taken and kept in dark until all the ASP changed back to SMEH. The remaining SMEH was measured to determine the unassembled ASP (SMEH) in the solution. As shown in the UV-Vis spectra in Supplementary Fig. 1, the percentage of assembled ASP was 91.66% according to the standard calibration curve in Supplementary Fig. 2b.

UV-Vis measurements of reversibility between ASP-CS transient assemblies and SMEH-CS solution. Spectrum and kinetics modes of UV-Vis were firstly used for the measurements. For the light-induced processes from SMEH-CS to ASP-CS, the spectrum mode was used at the fastest scan speed (high scan speed, one point every 5 nm) in order to avoid the thermal relaxation of ASP during the scanning. All the time of sampling and scanning was about 30 s. Therefore, when we measure the light-induced assembly process at a relative high temperature (30 ºC and 35 ºC), the thermal relaxation caused dissociation during the 30 s should influence the measurement to

some extent. The kinetics mode was used for scanning the thermal dissociation processes. The 424 nm absorbance and 650 nm transmittance of the samples were measured soon (about 5 s) after irradiation for 50 s.

Fluorescence measurements of reversibility between ASP-CS transient assemblies and SMEH-CS solution. Wavelength scan and time scan modes were both used for fluorescence variation of dissipative assemblies. For the light-induced processes from SMEH-CS to ASP-CS, the spectrum mode was used at the fastest scan speed (12000 nm/min) in order to avoid the thermal relaxation of ASP during the scanning. All the time of sampling and scanning was about 10 s. Therefore, when we measure the light-induced assembly process at a relative high temperature (30 ºC and 35 ºC), the thermal relaxation caused dissociation during the 10 s should influence the measurement to some extent. The kinetics mode was used for scanning the thermal dissociation processes. The fluorescence of the samples was measured soon (about 5 s) after irradiation for 50 s.

Observation of the formation and dissociation of the ASP-CS dissipative assembly from laser scanning confocal microscope after loading C153.[2] C153 was firstly dissolved in DMSO (the concentration is 10 mM). Then 0.3 μL of the C153 solution was added into 3 mL SMEH-CS aqueous solution to form SMEH-CS-C153 solution. ($[SMEH]_{initial}$ = 0.15 mM, [CS] = 40 μg/mL, [C153] = 0.001 mM) Fluorescence confocal images were collected of the SMEH-CS-C153 solution. The sample was kept in dark for three hours. The dynamics of assembly formation and dissociation was followed by collecting a time-series of images at the confocal microscope with temporal intervals of 5s under 420nm UV irradiation and 5min in dark. Fluorescence images were analyzed with ImageJ-Fiji software in order to count the particle numbers in each image and also to make a video from the time-series. The video of confocal images and the graph reporting the time evolution of the particle numbers were animated with Adobe Premiere software. The time-series stack has been analyzed using the selected parameters to extract the particle count from each image. The background threshold level has been set to 10-255 (for 8-bit images) and the minimum object dimension has been set to 3 pixel. Discarding the data set at the lowest threshold and size, the amplitude of the second peak is approximately 70% of the first peak one. More remarkably, for all analysis configurations, the amplitude of highest peak is 50 times than the corresponding control mean value, confirming the relevance of such signature with respect to the background noise.

Determination of the half-lives from the time-dependent variation of UV-Vis absorbance and fluorescence emission. The half-lives of absorbance were determined by reading the time that SMEH reached the average absorbance of the initial and the end state in SMEH or SMEH-CS solution. The half-lives of fluorescence were determined by reading the time that the fluorescence emission reached the average emission of the initial and the end state during the fluorescence variation.

Supplementary Movies. Supplementary Movie 1 showed the confocal images of light-induced formation of ASP-CS transient assemblies and its spontaneous dissociation in the dark by loading C153 as well as total number of fluorescent particle counts detected over time. Supplementary Movie 2 and 3 showed dynamic fluorescent variation of

loaded TCPE over time during light-induced assembly and thermal dissociation process, respectively. In Supplementary Movie 2, the irradiation light (420 nm) was on the left side. In Supplementary Movie 2 and 3, there is a weak excitation light (365 nm) on the right side. The environment temperature was ~25 ºC.

B. Reversible photo-isomerization between SMEH and ASP

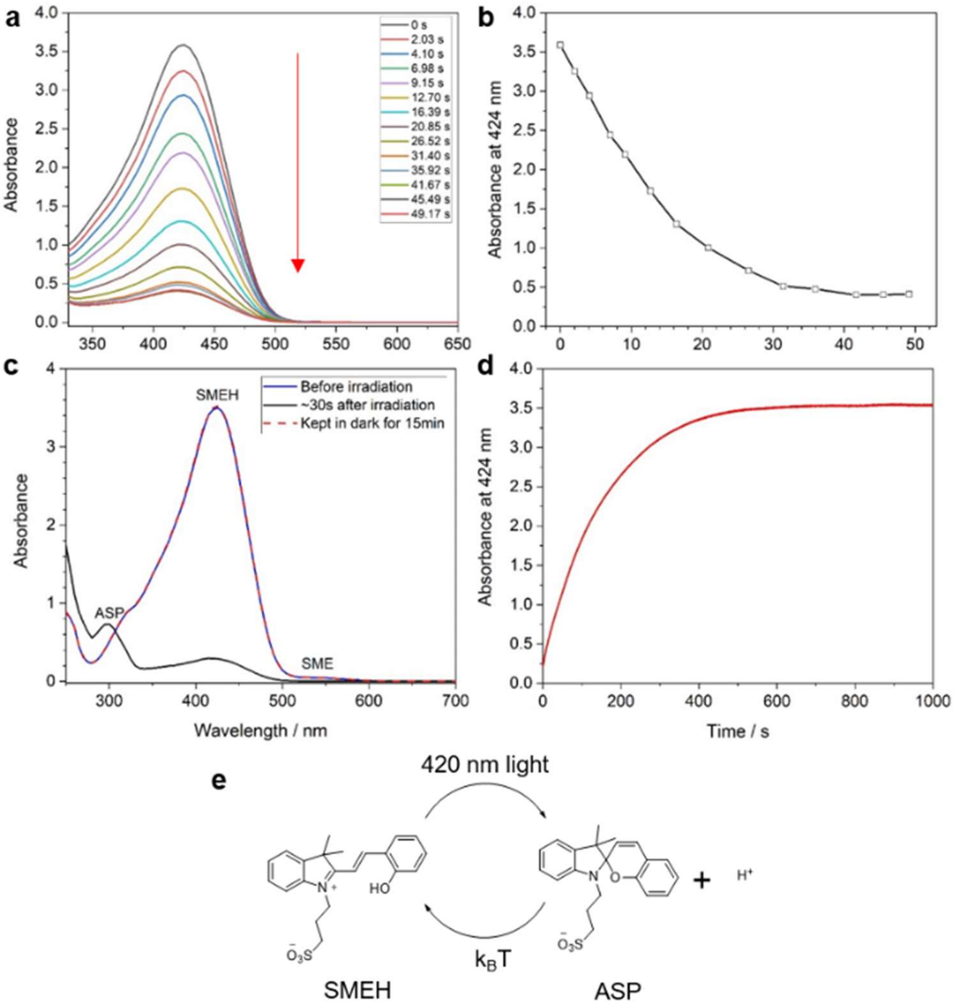

Supplementary Figure 1. **a,** UV-Vis absorbance spectra of SMEH (0.15 mM) in aqueous solution upon irradiation with different time length (0 s – 49.17 s). **b,** Absorbance variation at 424 nm of a). **c,** UV-Vis absorbance spectra of SMEH (0.15 mM) in aqueous solution before irradiation, ~30 s (time between the end of irradiation and the end of UV-Vis measurement) and kept in dark for 15 min. **d,** Variation of absorbance at 424 nm of SMEH after irradiation for 50 s and kept in dark at 25 ºC. **e,** Isomerization of SMEH to ASP form after irradiated with 420 nm light, and then transform to SMEH form spontaneously when being in dark.

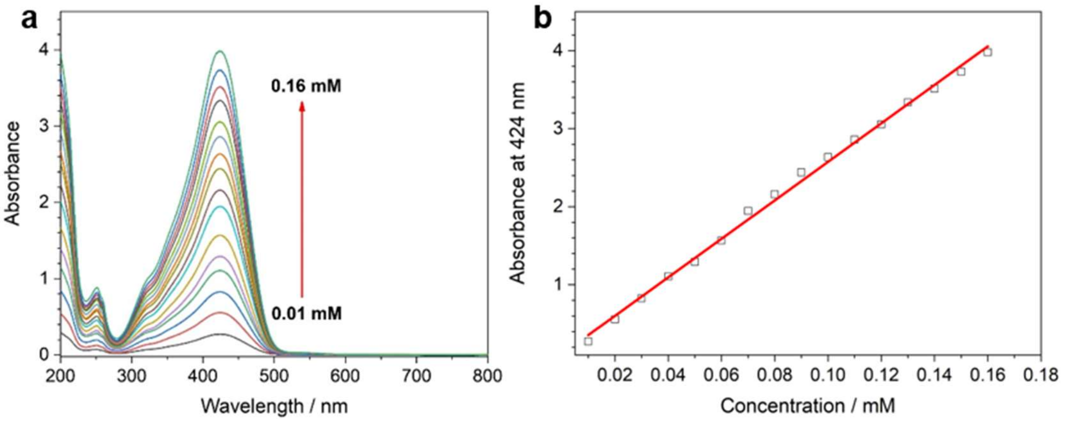

Supplementary Figure 2. a, UV-Vis absorbance spectra of SMEH on increasing in concentration (0.01 mM – 0.16 mM) in aqueous solution. **b,** Calibration curve of SMEH showing the relationship between concentration of SMEH and absorbance at 424 nm.

The calibration equation in **b** is: $A(424\ \text{nm}) = 0.1084 + 22.66074 \times c(\text{SMEH}), R^2 = 0.997$

C. Determination of the preferable assembly concentration of ASP-CS by optical transmittance[3]

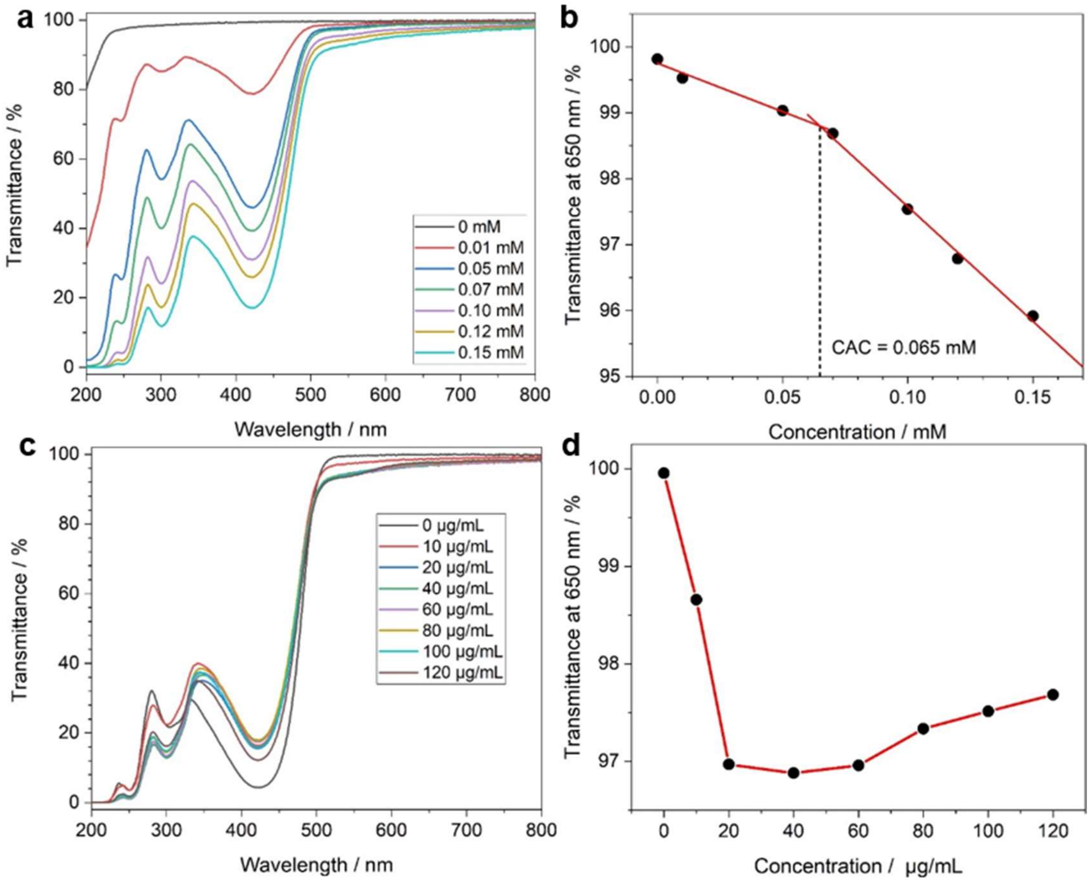

Supplementary Figure 3. a, Transmittance of CS (60 μg/mL) in varying concentration of ASP from 0 to 0.15 mM right after irradiation with 420 nm light for 50 s. **b,** Transmittance at 650 nm of **a**. **c,** Transmittance of ASP (0.15 mM) in varying concentration of CS (0 – 120 μg/mL) right after irradiation. **d,** Transmittance at 650 nm of **c**.

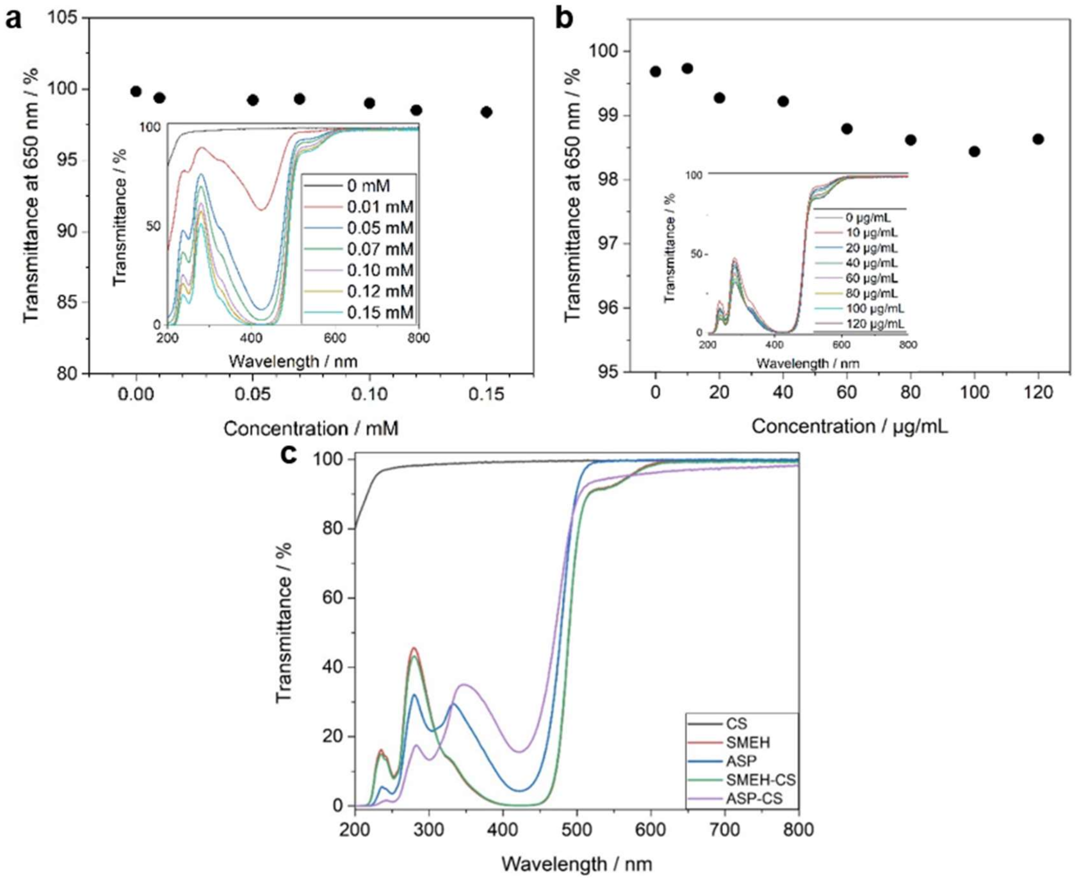

Supplementary Figure 4. a, Dependence of the transmittance of CS (60 μg/mL) in varying concentration of SMEH from 0 to 0.15 mM at 650 nm without irradiation. Inset: corresponding transmittance spectrum. **b,** Dependence of transmittance of SMEH (0.15 mM) in varying concentration of CS (0 – 120 μg/mL) at 650 nm without irradiation. Inset: corresponding transmittance spectrum. **c,** Transmittance of CS (40 μg/mL), SMEH (0.15 mM), ASP (0.15 mM), SMEH (0.15 mM)-CS (40 μg/mL), and ASP (0.15 mM)-CS (40 μg/mL), respectively.

D. Formation and Dissociation of dissipative SMEH-CS system

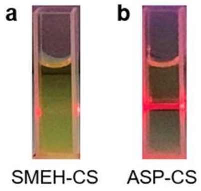

Supplementary Figure 5. Tyndall effect of **a** SMEH-CS and **b** ASP-CS in aqueous solution. ($[SMEH]_{initial}$ = 0.15 mM, [CS] = 40 μg/mL)

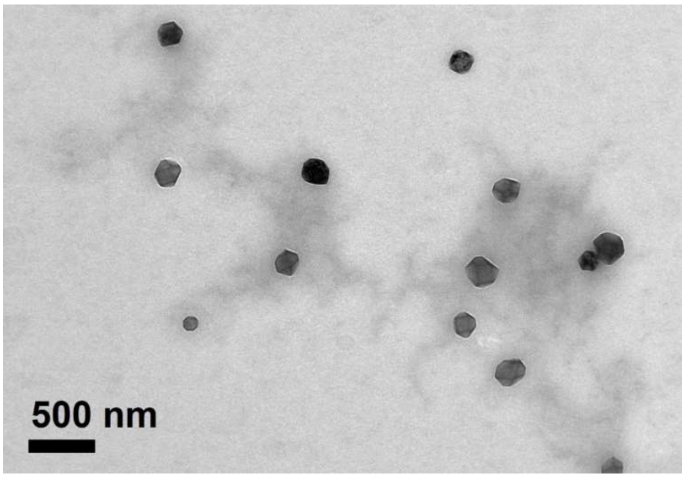

Supplementary Figure 6. a, Amplified TEM image ASP-CS polyhedron nanoparticles.

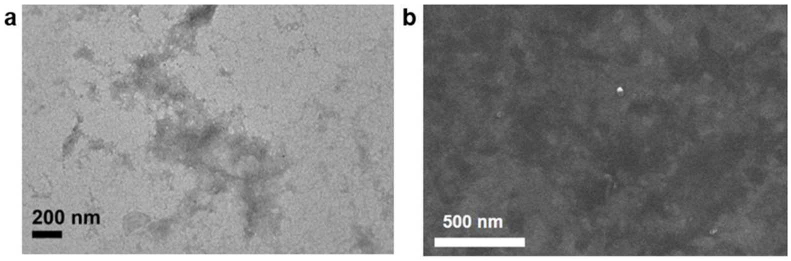

Supplementary Figure 7. a, TEM image and **b** SEM image of SMEH (0.15 mM)-CS (40 µg/mL) in the dark.

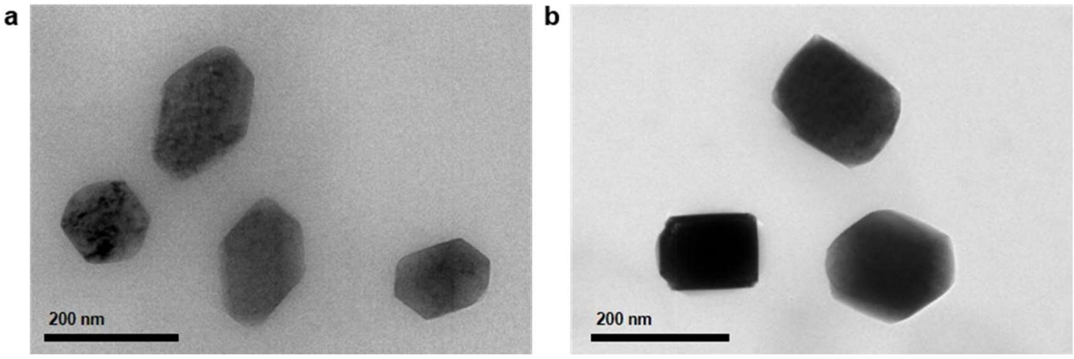

Supplementary Figure 8. TEM images of ASP-CS transient nanoparticles at **a** 15 °C and **b** 35 °C.

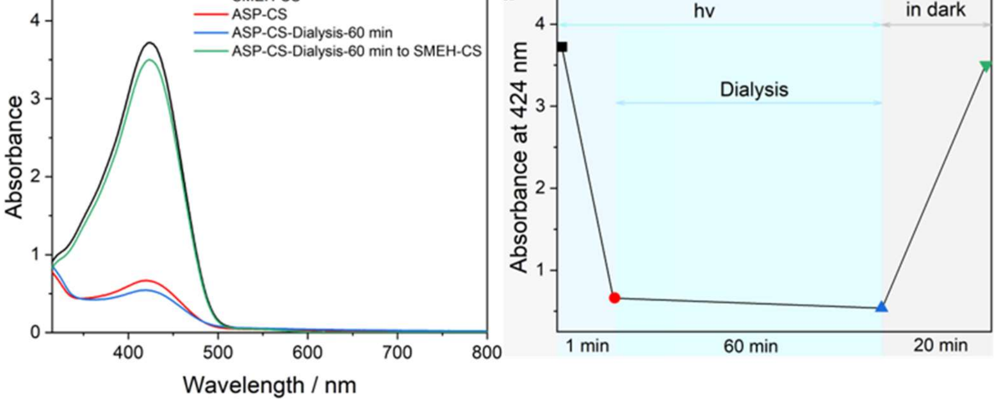

Supplementary Figure 9. a, UV-Vis absorption of SMEH-CS, ASP-CS, ASP-CS right after dialysis for 1 h, SMEH-CS from the dialyzed ASP-CS in dark. **b,** absorbance at 424 nm of **a** towards the time during the irradiation, dialysis and keeping in dark.

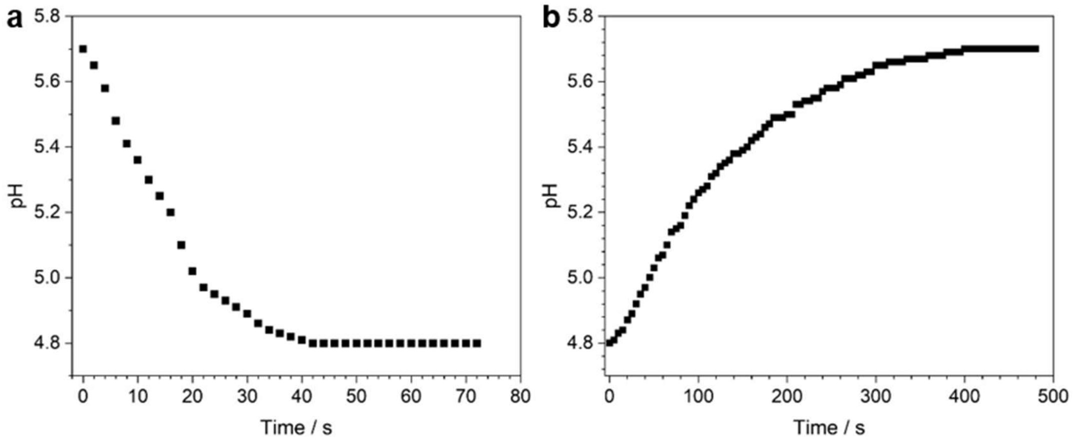

Supplementary Figure 10. a, pH decreasing process of SMEH-CS upon irradiation in aqueous solution. **b,** pH increasing process of ASP-CS in the dark in aqueous solution right after irradiation. ($[SMEH]_{initial}$ = 0.15 mM, [CS] = 40 μg/mL).

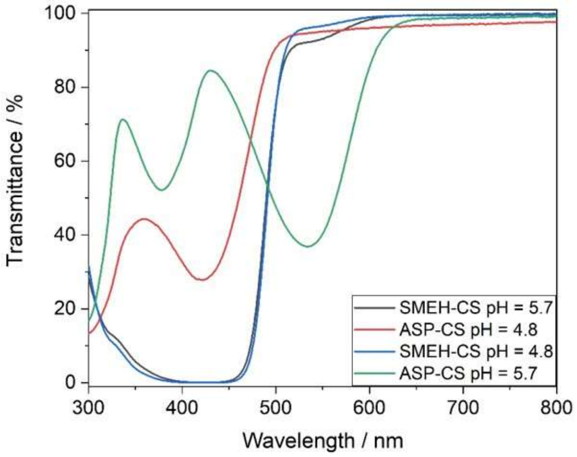

Supplementary Figure 11. Transmittance of aqueous solution containing SMEH-CS (pH = 5.7), ASP-CS (pH = 4.8), SMEH-CS (pH = 4.8) and ASP-CS (pH = 5.7), respectively. ($[SMEH]_{initial}$ = 0.15 mM, [CS] = 40 μg/mL).

E. Characterization of reversibility and dissipative kinetics of SMEH-CS system

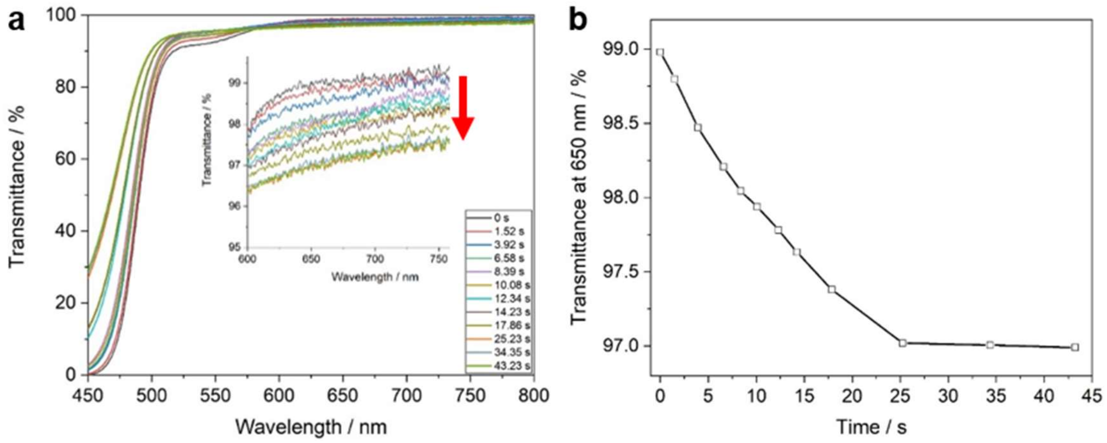

Supplementary Figure 12. a, Transmittance of SMEH-CS versus irradiation time in aqueous solution. **b,** Transmittance at 650 nm of **a**. ([SMEH]$_{initial}$ = 0.15 mM, [CS] = 40 μg/mL).

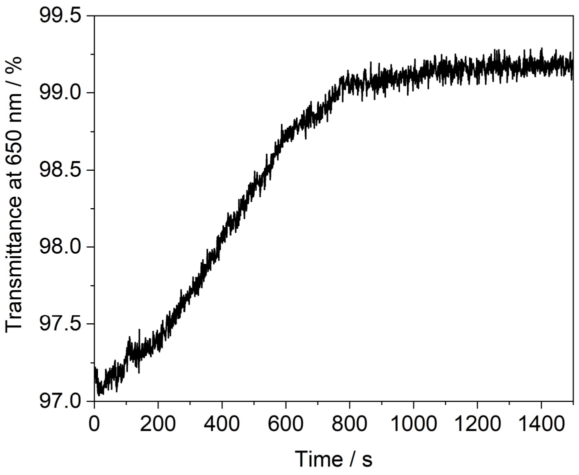

Supplementary Figure 13. Kinetic process of transmittance at 650 nm from ASP-CS to SMEH-CS in aqueous solution kept in dark at 25 °C. ([SMEH]$_{initial}$= 0.15 mM, [CS] = 40 μg/mL).

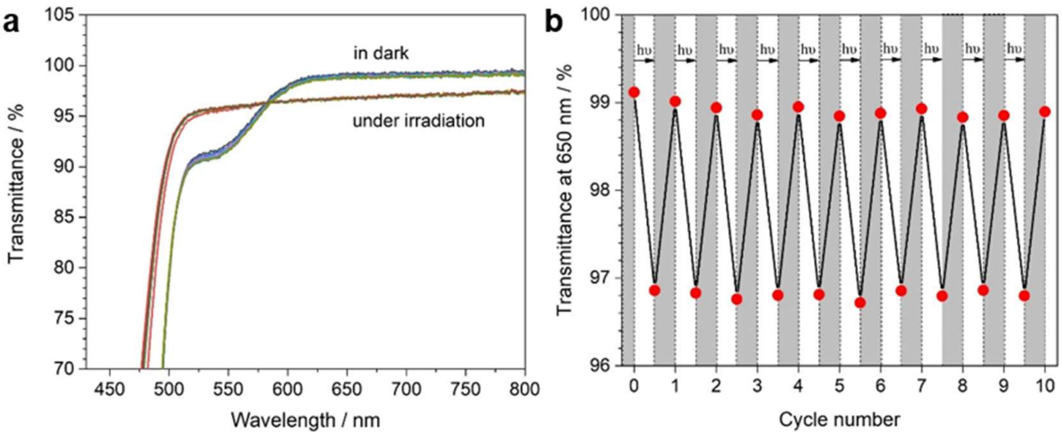

Supplementary Figure 14. a, Transmittance changing cycles of SMEH-CS in aqueous solution after 50 s of irradiation under 420 nm light and kept in dark for 20 min at 25 °C. **b,** Changing cycles of transmittance at 650 nm of **a**. ([SMEH]$_{initial}$ = 0.15 mM, [CS] = 40 μg/mL).

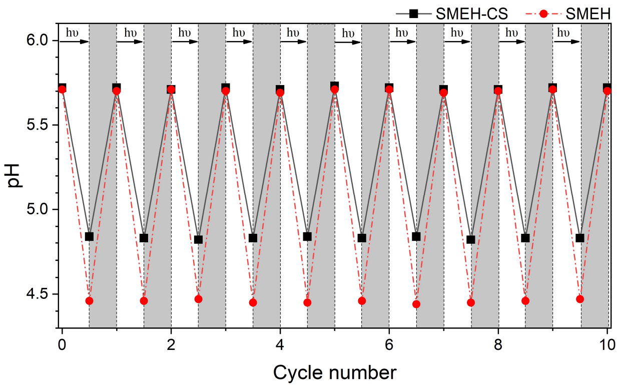

Supplementary Figure 15. Comparison of pH changing cycles between SMEH-CS and only SMEH in aqueous solution after 50 s of irradiation under 420 nm light and kept in dark for 20 min at 25 °C. ([SMEH]$_{initial}$ = 0.15 mM, [CS] = 40 μg/mL).

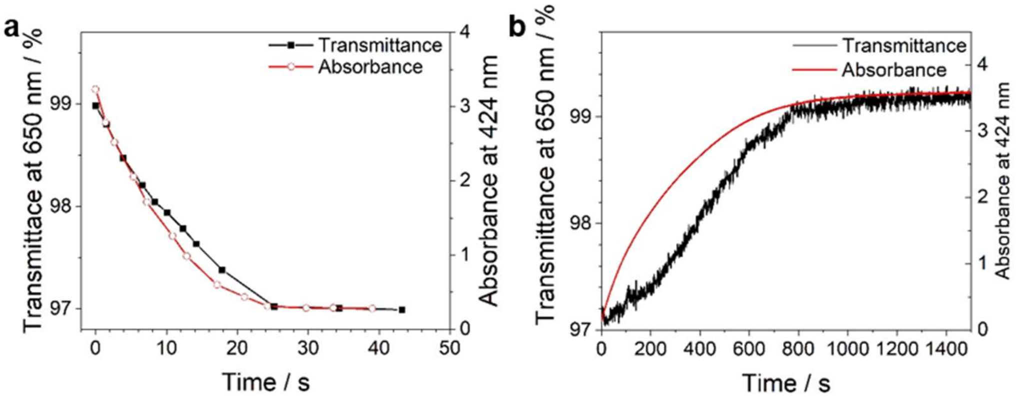

Supplementary Figure 16. a, Co-ordinance of decreasing process between transmittance at 650 nm and absorbance at 424 nm of SMEH-CS in aqueous solution over irradiation time. **b,** Co-ordinance of recovery process between transmittance at 650 nm and absorbance at 424 nm of SMEH-CS in aqueous solution after 50 s of irradiation and kept in dark at 25 ºC. ([SMEH]$_{initial}$ = 0.15 mM, [CS] = 40 μg/mL).

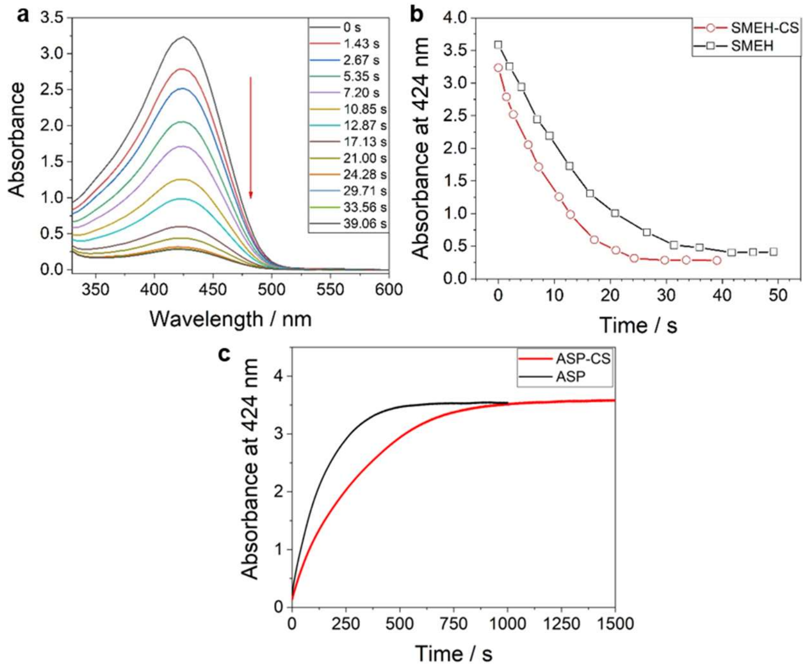

Supplementary Figure 17. a, UV-Vis absorbance spectra of SMEH-CS in aqueous solution upon irradiation by 420 nm light with different time length (0 s – 39.06 s). **b,** Comparison of absorbance at 424 nm decreasing process during irradiation between SMEH-CS and SMEH alone in aqueous solution. **c,** Comparison of absorbance at 424 nm increasing kinetics right after 50 s of irradiation and kept in dark at 25 ºC between aqueous solution of ASP-CS and ASP alone in solution. ([SMEH]$_{initial}$ = 0.15 mM, [CS] = 40 μg/mL)

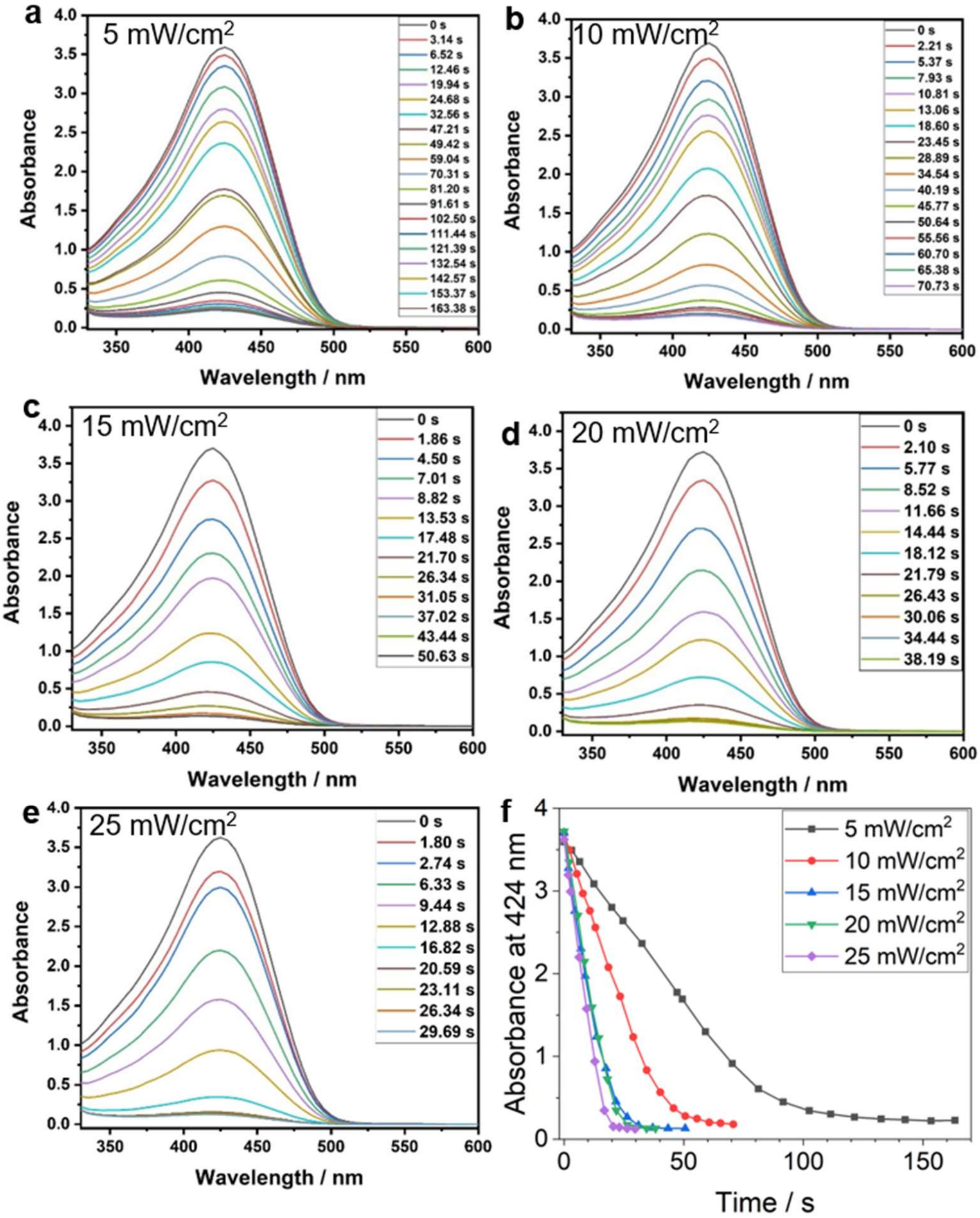

Supplementary Figure 18. a-e, UV-Vis absorbance spectra of SMEH in aqueous solution upon irradiation by 420 nm light with different power density (5 mW/cm^2, 10 mW/cm^2, 15 mW/cm^2, 20 mW/cm^2 and 25 mW/cm^2, respectively) at 15 ^{o}C. **f,** Decreasing process of UV-Vis absorbance at 424 nm of (**a-e**). ([SMEH]$_{initial}$ = 0.15 mM).

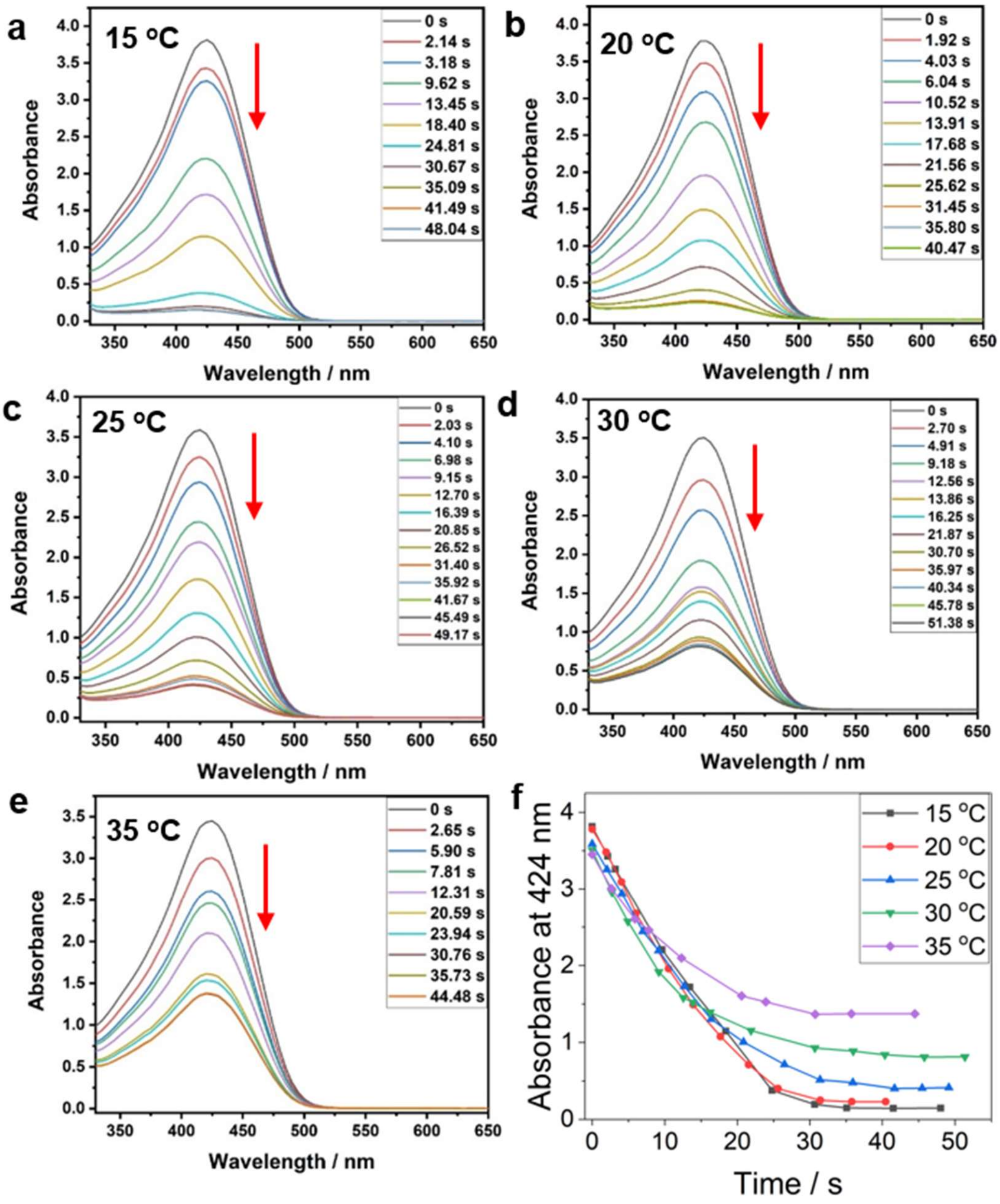

Supplementary Figure 19. a-e, UV-Vis absorbance spectra of SMEH in aqueous solution upon irradiation by 420 nm light with different time length at different temperature(15 °C, 20 °C, 25 °C , 30 °C and 35 °C). **f,** Decreasing process of UV-Vis absorbance at 424 nm of (**a-e**). ([SMEH]$_{initial}$ = 0.15 mM).

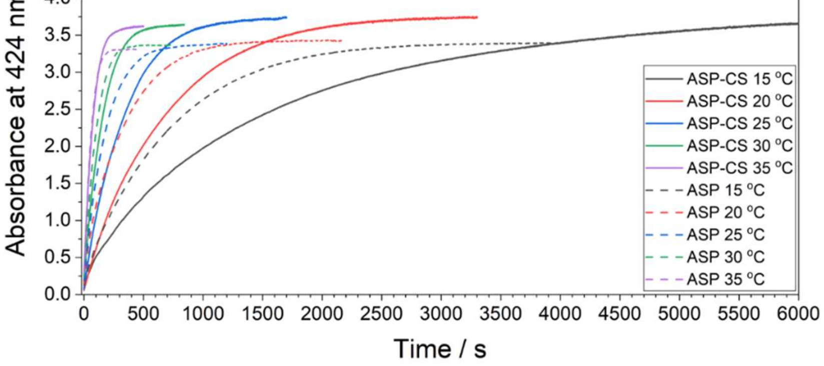

Supplementary Figure 20. Comparison of absorbance at 424 nm increasing kinetics right after 50 s of irradiation and kept in the dark between aqueous solution of ASP-CS and ASP alone in solution at different temperature (15 ºC, 20 ºC, 25 ºC, 30 ºC and 35 ºC respectively). ($[SMEH]_{initial}$ = 0.15 mM, [CS] = 40 μg/mL).

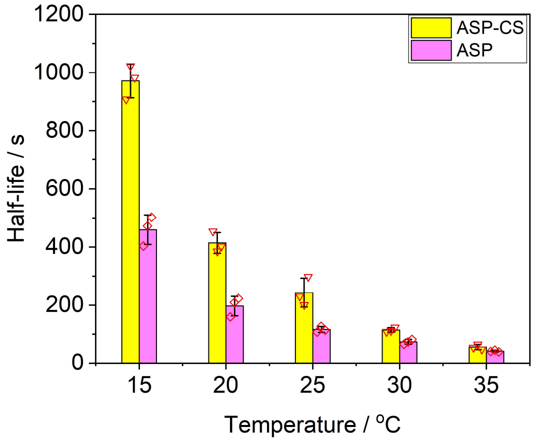

Supplementary Figure 21. Comparison of thermal dissociation half-lives at different temperature from 15 ºC to 35 ºC detected on the UV-Vis absorbance at 424 nm of ASP-CS and ASP. ($[SMEH]_{initial}$ = 0.15 mM, [CS] = 40 μg/mL). n = 3 independent experiments, with the bar data indicating mean ± SD.

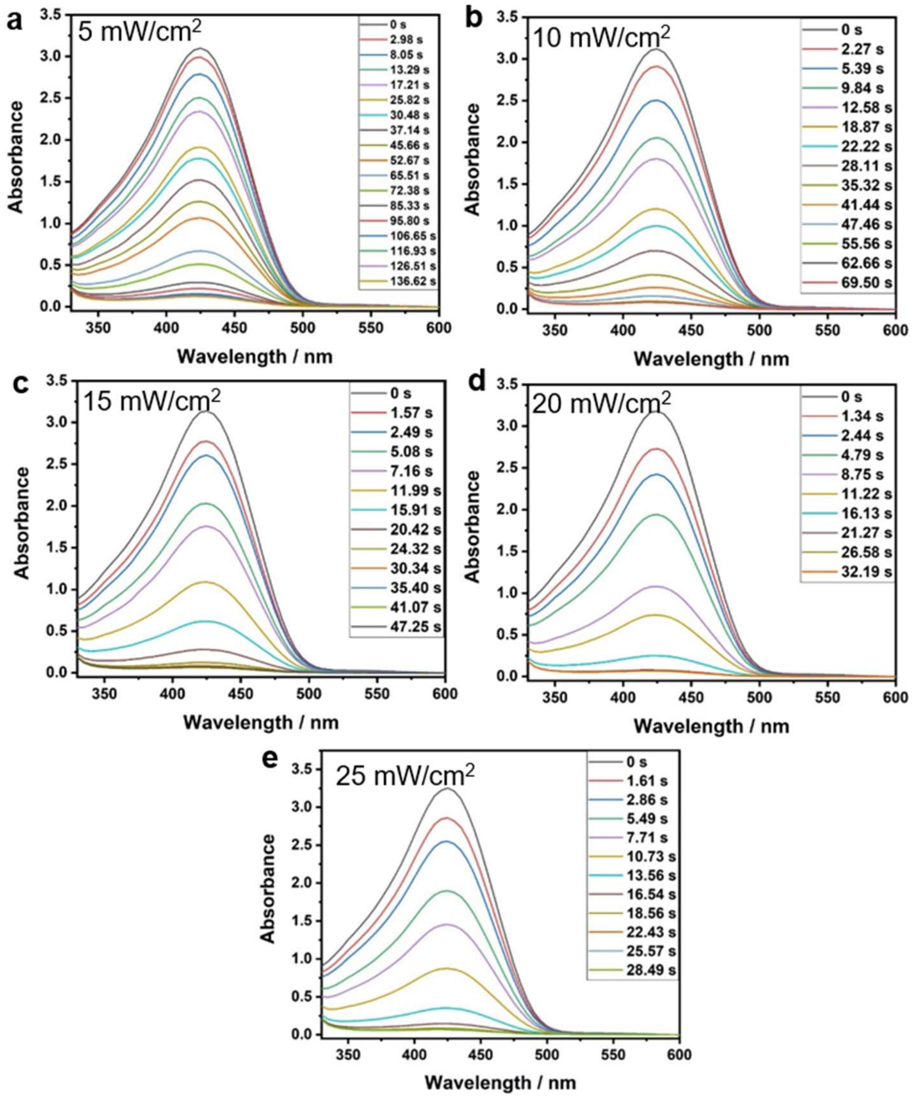

Supplementary Figure 22. a-e, UV-Vis absorbance spectra of SMEH-CS in aqueous solution upon irradiation by 420 nm light with different power density (5 mW/cm^2, 10 mW/cm^2, 15 mW/cm^2, 20 mW/cm^2 and 25 mW/cm^2, respectively) at 15 ºC. ([SMEH]$_{initial}$ = 0.15 mM, [CS] = 40 μg/mL)

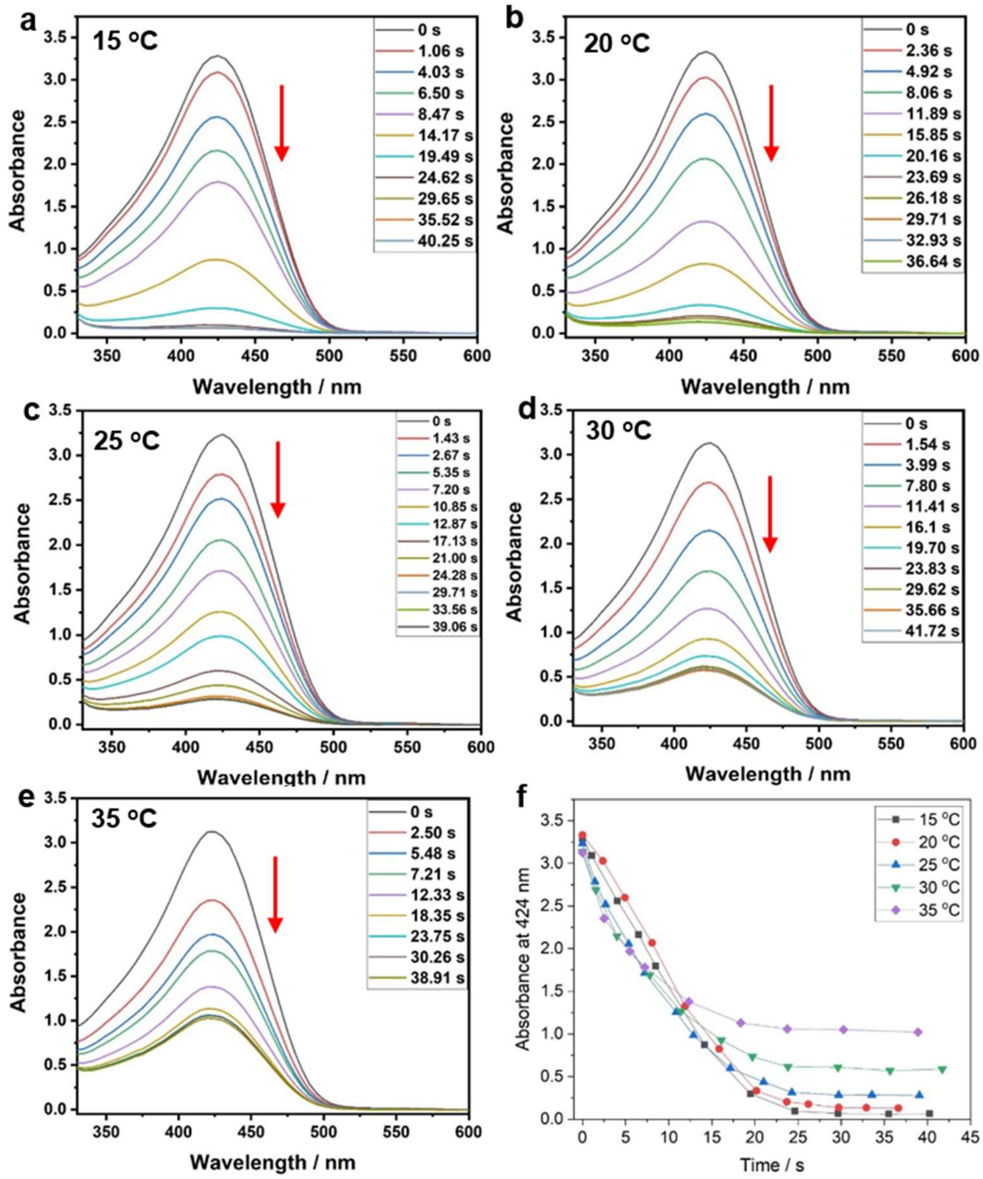

Supplementary Figure 23. a-e, UV-Vis absorbance spectra of SMEH-CS in aqueous solution upon irradiation by 420 nm light (optical power density: 15 mW/cm^2) with different time length at different temperature (15 ºC, 20 ºC, 25 ºC , 30 ºC and 35 ºC, respectively). **f,** Decreasing process of UV-Vis absorbance at 424 nm of (**a-e**). ([SMEH]$_{initial}$ = 0.15 mM, [CS] = 40 μg/mL)

F. Chemical structure of fluorophores in this work

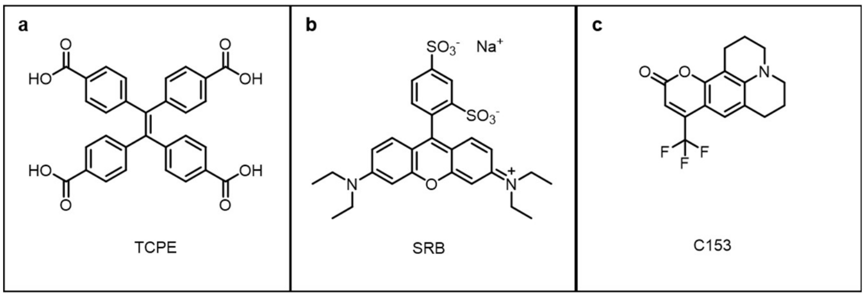

Supplementary Figure 24. Chemical structure of **a,** TCPE (tetra(4-carboxyphenyl)ethylene), **b,** SRB (sulforhodamine B) and **c,** C153 (coumarin C153) used as fluorophores in this work.

G. Demonstration of formation and dissociation process of the dissipative self-assemblies by loading C153

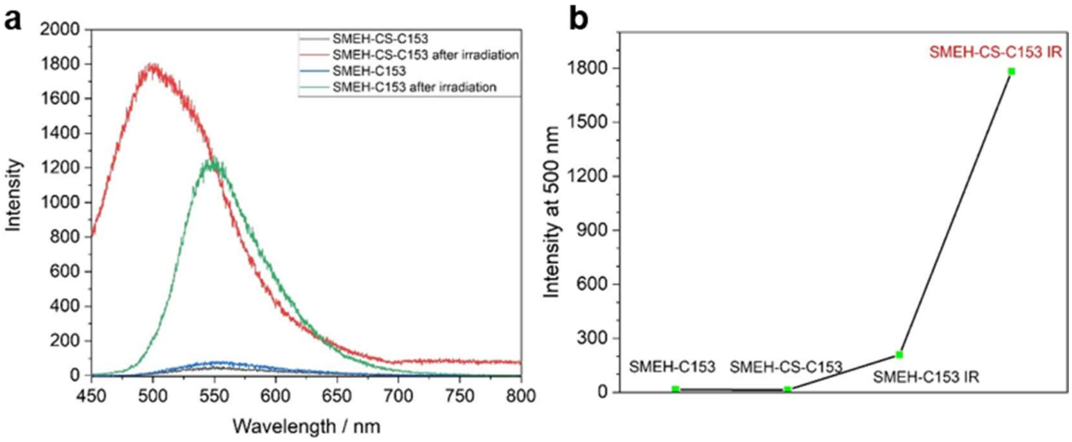

Supplementary Figure 25. a, Fluorescence intensity of SMEH-CS-C153 before and after irradiation by 420 nm light (optical power density: 15 mW/cm^2) at 25 ^{o}C in aqueous solution, SMEH-C153 before and after irradiation at the same condition, respectively. **b,** Intensity at 500 nm of **a**. ([SMEH]$_{initial}$ = 0.15 mM, [CS] = 40 µg/mL, [C153] = 0.001 mM, λ_{ex} = 425 nm)

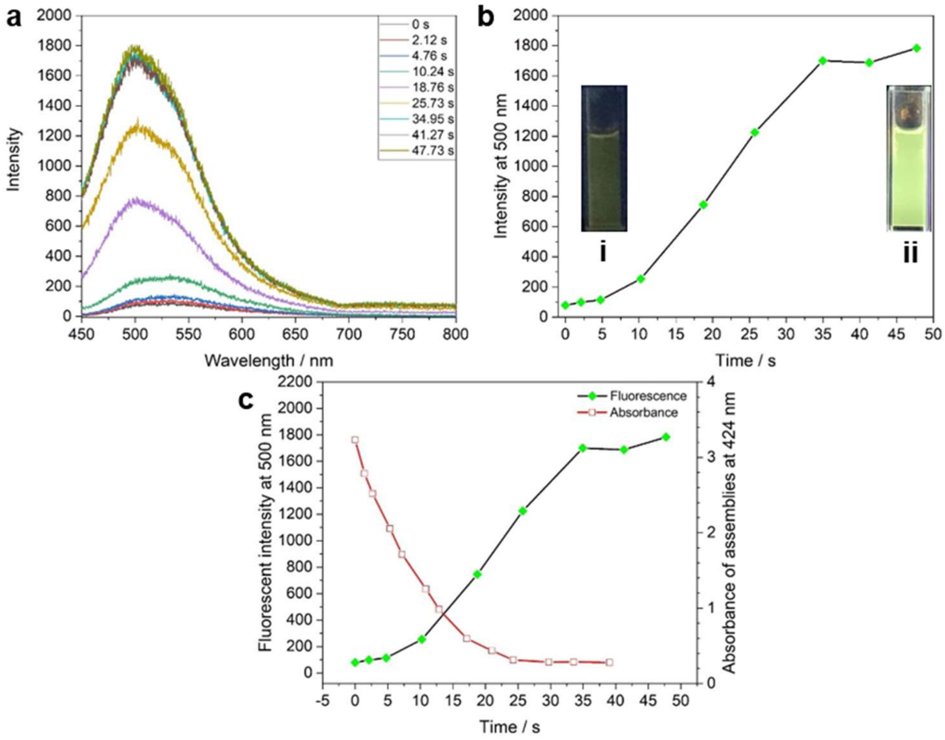

Supplementary Figure 26. a, Fluorescence spectra of SMEH-CS-C153 upon irradiation by 420 nm light (optical power density: 15 mW/cm^2) at 25 °C in aqueous solution. **b,** Intensity at 500 nm of a. (Inset: photographs of SMEH-CS-C153 in dark (**i**) and right after irradiation by 420 nm light (**ii**) under 365 nm UV lamp). **c,** co-ordinance of fluorescent intensity at 500 nm increasing process upon irradiation with absorbance of assemblies (SMEH-CS) at 424 nm. ([SMEH]$_{initial}$ = 0.15 mM, [CS] = 40 μg/mL, [C153] = 0.001 mM, λ_{ex} = 425 nm)

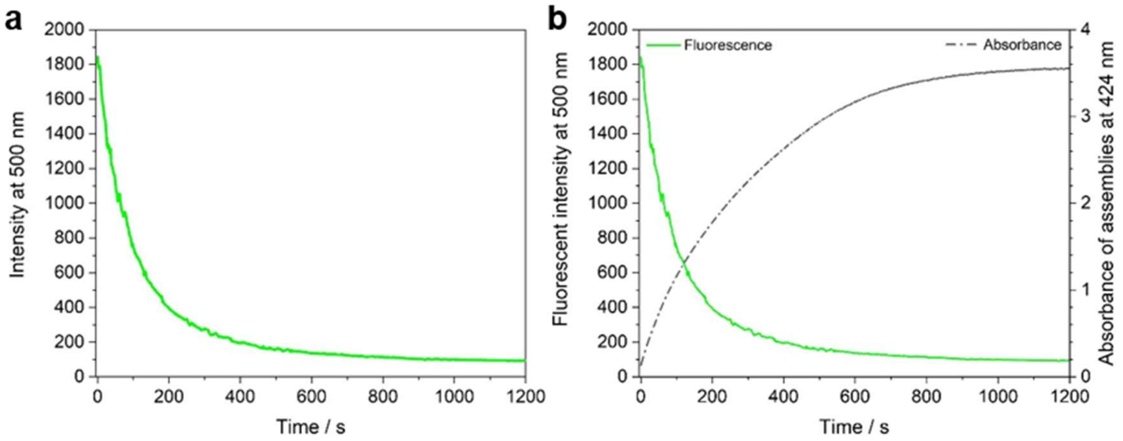

Supplementary Figure 27. a, Decreasing kinetics of fluorescence intensity at 500 nm of SMEH-CS-C153 after irradiation by 420 nm light (optical power density: 15 mW/cm^2) for 50 s at 25 °C in aqueous solution and kept in dark. **b,** Co-ordinance of fluorescence intensity at 500 nm decreasing process after irradiation with absorbance of assemblies (SMEH-CS) right after irradiation at 424 nm. ([SMEH]$_{initial}$ = 0.15 mM, [CS] = 40 μg/mL, [C153] = 0.001 mM, λ_{ex} = 425 nm)

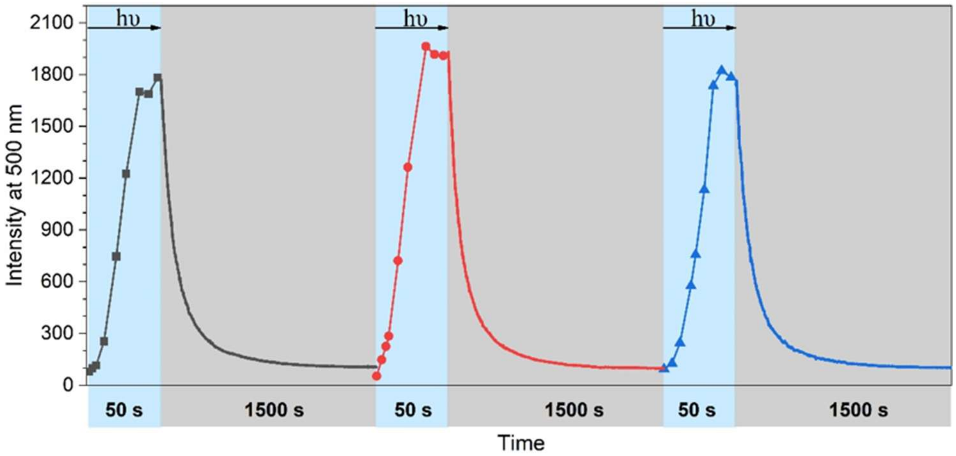

Supplementary Figure 28. Fluorescence intensity at 500 nm changing cycles of SMEH-CS-C153 in aqueous solution during 50 s of irradiation by 420 nm light (optical power density: 15 mW/cm^2) and decreasing kinetics when kept in dark right after irradiation at 25 °C. ([SMEH]$_{initial}$ = 0.15 mM, [CS] = 40 μg/mL, [C153] = 0.001 mM, λ_{ex} = 425 nm)

H. Confocal images of the dissipative self-assemblies by loading C153

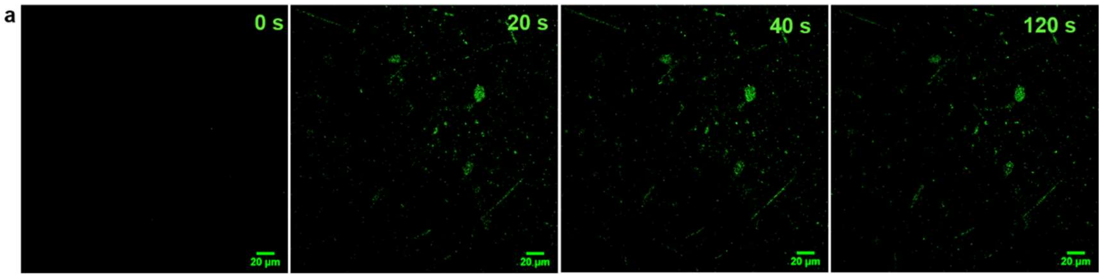

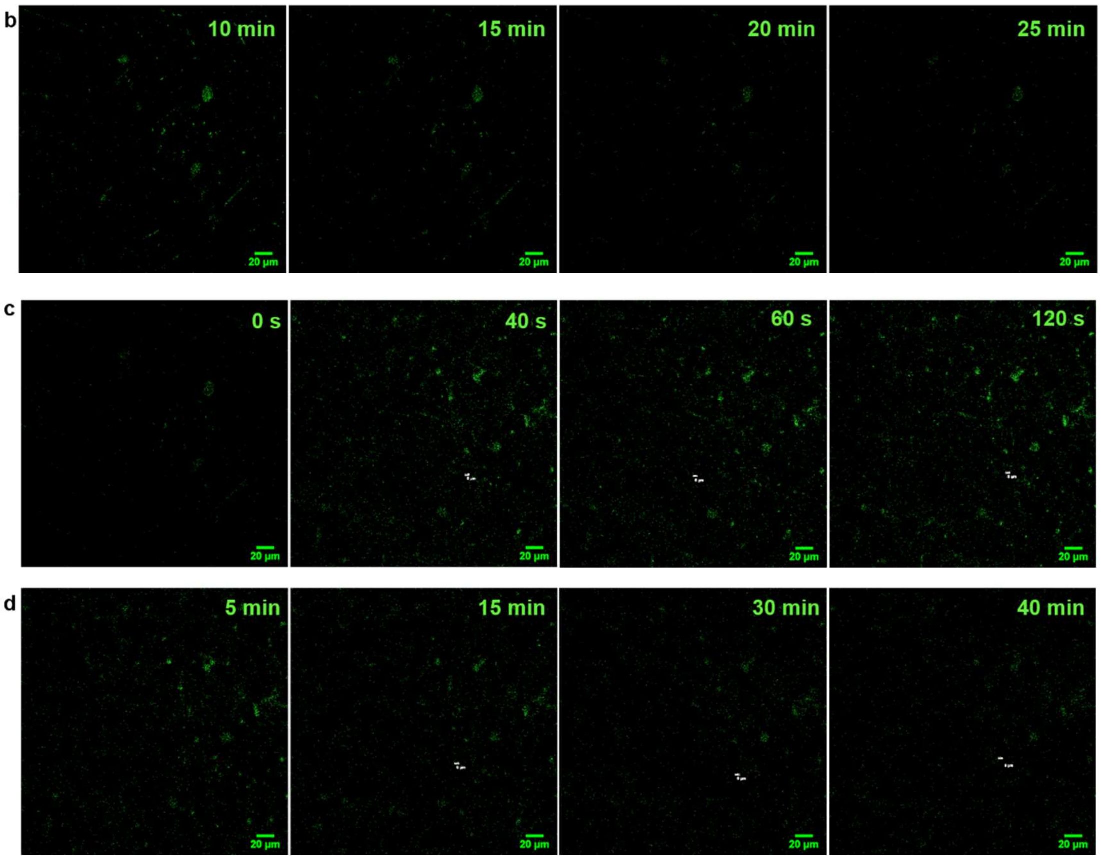

Supplementary Figure 29. Confocal images of SMEH-CS-C153 in aqueous solution over time showing reversible formation/dissociation of fluorophore bound nanoparticles, including 2 cycles. **a,** the 1st cycle of irradiation process over time showing the increasing number of assemblies. **b,** the 1st cycle of disassembly process over time after irradiation and kept in dark. **c,** the 2nd cycle of irradiation process over time showing the increasing number of assemblies. **d,** the 2nd cycle of disassembly process in dark, reflecting decreasing number of nanoparticles keeping in dark. Experimental details: ($[SMEH]_{initial}$ = 0.15 mM, [CS] = 40 μg/mL, [C153] = 0.001 mM, λ_{ex} = 405 nm, λ_{em} = 480 – 520 nm)

I. Demonstration of time-dependent AIE and ACQ processes by loading different fluorophores

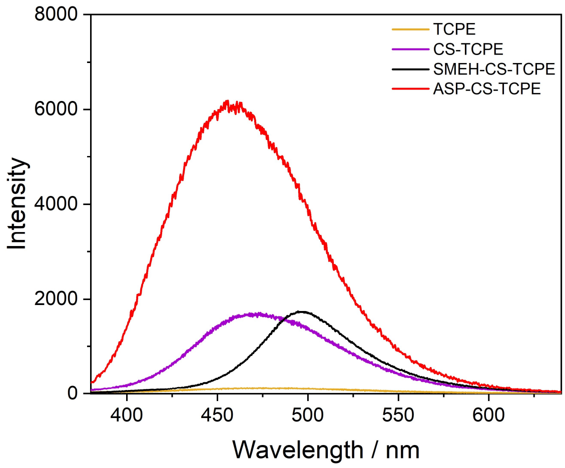

Supplementary Figure 30. Fluorescence spectra of TCPE, CS-TCPE, SMEH-CS-TCPE, ASP-CS-TCPE at 25 °C in aqueous solution. ($[SMEH]_{initial}$ = 0.15 mM, [CS] = 40 μg/mL, [TCPE] = 0.01 mM, λ_{ex} = 325 nm)

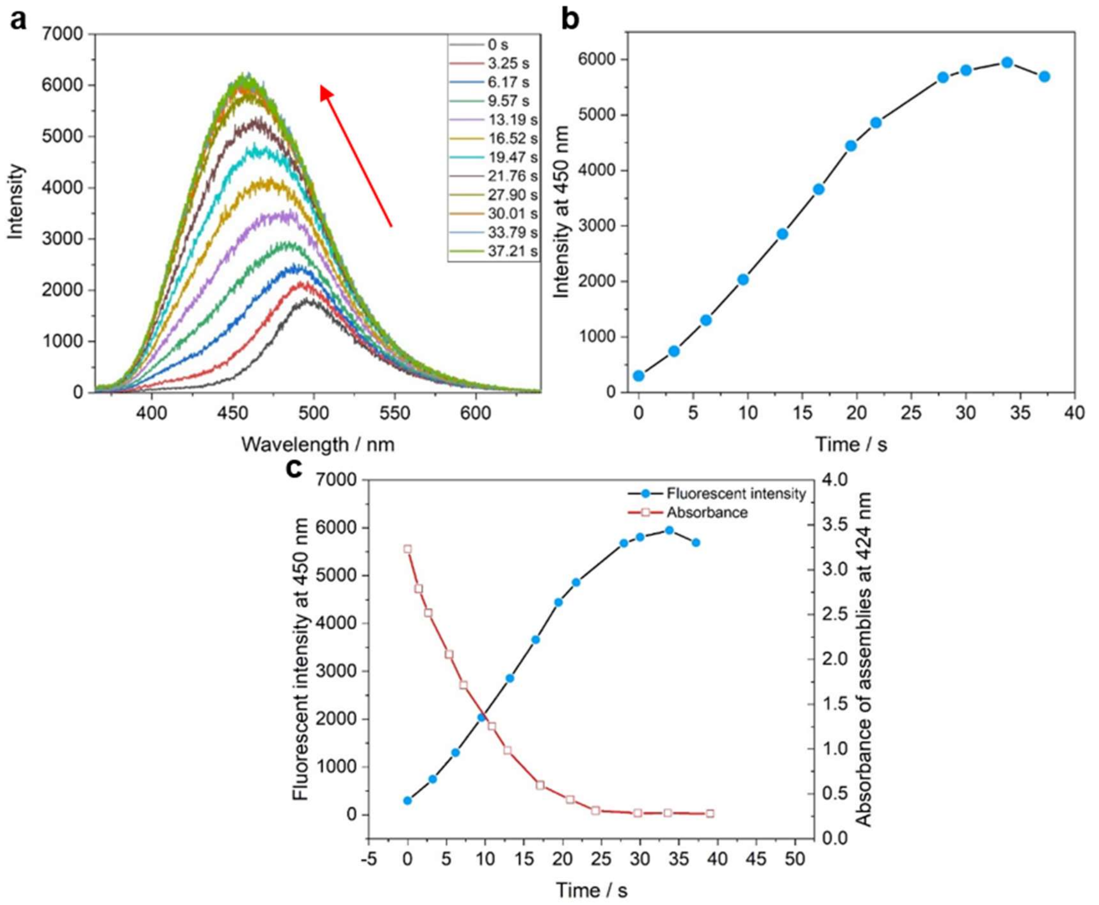

Supplementary Figure 31. a, Fluorescence spectra of SMEH-CS-TCPE upon irradiation by 420 nm light (optical power density: 15 mW/cm^2) at 25 °C in aqueous solution. **b,** Intensity at 450 nm of **a**. **c,** Co-ordinance of fluorescent intensity at 450 nm increasing process upon irradiation with absorbance of assemblies (SMEH-CS) at 424 nm. ([SMEH]$_{initial}$ = 0.15 mM, [CS] = 40 µg/mL, [TCPE] = 0.01 mM, λ_{ex} = 325 nm)

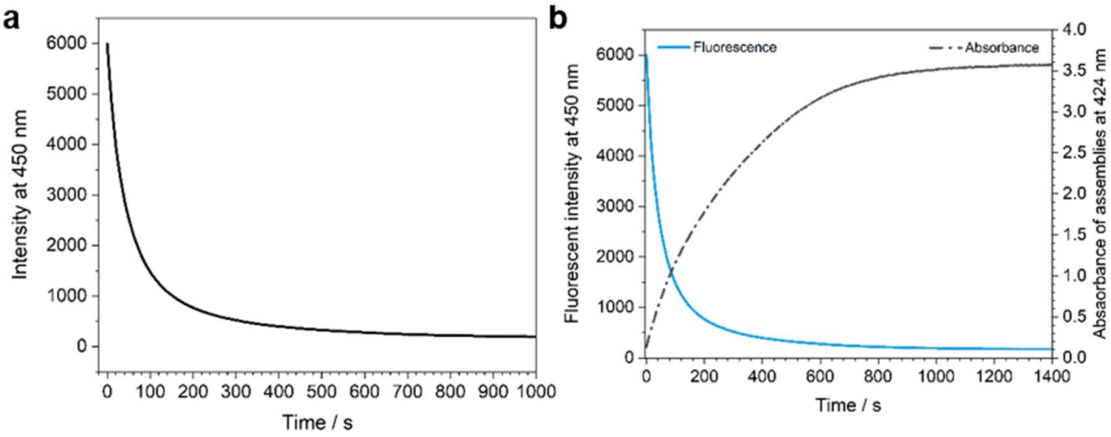

Supplementary Figure 32. a, Decreasing kinetics of fluorescence intensity at 450 nm of SMEH-CS-TCPE after irradiation by 420 nm light (optical power density: 15 mW/cm^2) for 50 s at 25 °C in aqueous solution and kept in dark. **b,** Co-ordinance of fluorescence intensity at 450 nm decreasing process after irradiation with absorbance of assemblies (SMEH-CS) after irradiation at 424 nm. ([SMEH]$_{initial}$ = 0.15 mM, [CS] = 40 µg/mL, [TCPE] = 0.01 mM, λ_{ex} = 325 nm)

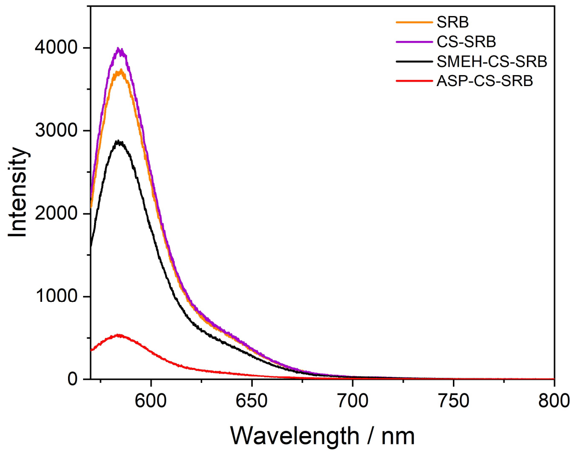

Supplementary Figure 33. Fluorescence spectra of SRB, CS-SRB, SMEH-CS-SRB and ASP-CS-SRB at 25 °C in aqueous solution. ([SMEH]$_{initial}$ = 0.15 mM, [CS] = 40 μg/mL, [SRB] = 0.001 mM, λ_{ex} = 560 nm)

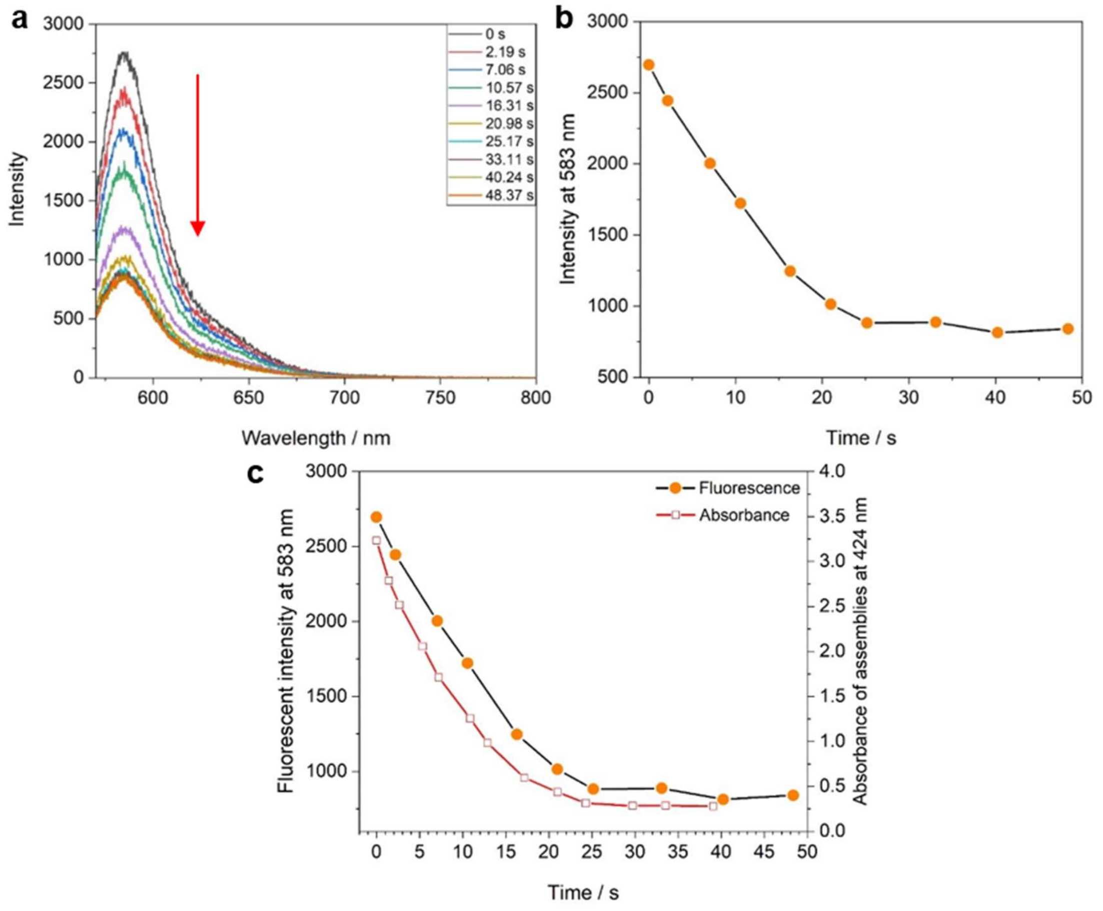

Supplementary Figure 34. a, Fluorescence spectra of SMEH-CS-SRB upon irradiation by 420 nm light (optical power density: 15 mW/cm^2) at 25 °C in aqueous solution. **b,** Intensity at 583 nm of **a**. **c,** Co-ordinance of fluorescent intensity at 583 nm decreasing process upon irradiation with absorbance of assemblies (SMEH-CS) at 424 nm. ([SMEH]$_{initial}$ = 0.15 mM, [CS] = 40 μg/mL, [SRB] = 0.001 mM, λ_{ex} = 560 nm)

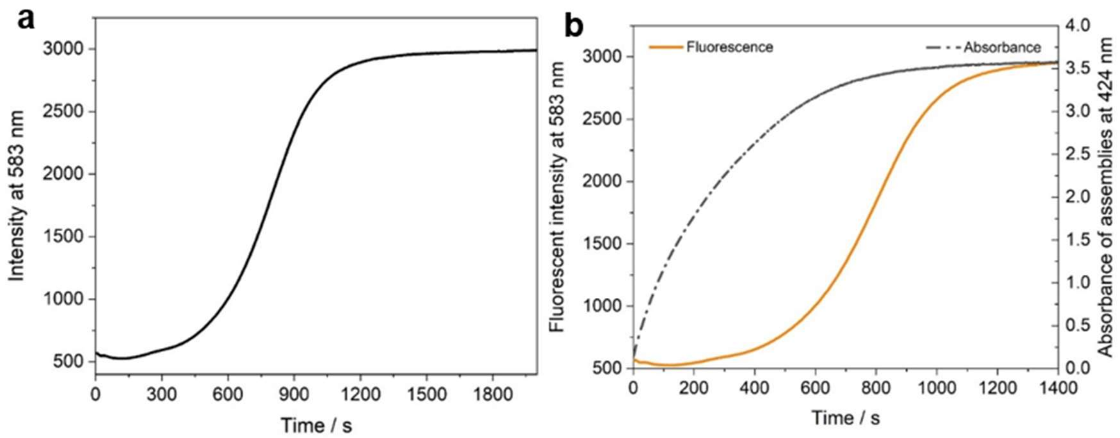

Supplementary Figure 35. a, Increasing kinetics of fluorescence intensity at 583 nm of SMEH-CS-SRB after irradiation by 420 nm light (optical power density: 15 mW/cm^2) for 50 s at 25 ºC in aqueous solution and kept in dark. **b,** Co-ordinance of fluorescence intensity at 450 nm increasing process after irradiation with absorbance of assemblies (SMEH-CS) after irradiation at 424 nm. ([SMEH]$_{initial}$ = 0.15 mM, [CS] = 40 μg/mL, [TCPE] = 0.01 mM, λ_{ex} = 560 nm)

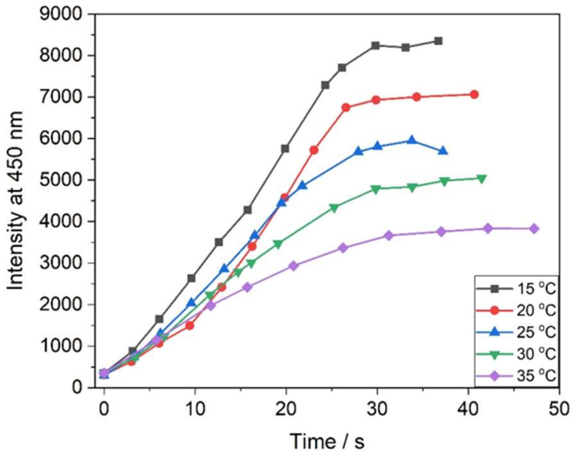

Supplementary Figure 36. Fluorescence intensity at 450 nm of SMEH-CS-TCPE in aqueous solution as a function of irradiation (420 nm light, optical power density: 15 mW/cm^2) time at different temperature (15 ºC, 20 ºC, 25 ºC, 30 ºC and 35 ºC, respectively). ([SMEH]$_{initial}$ = 0.15 mM, [CS] = 40 μg/mL, [TCPE] = 0.01 mM, λ_{ex} = 325 nm)

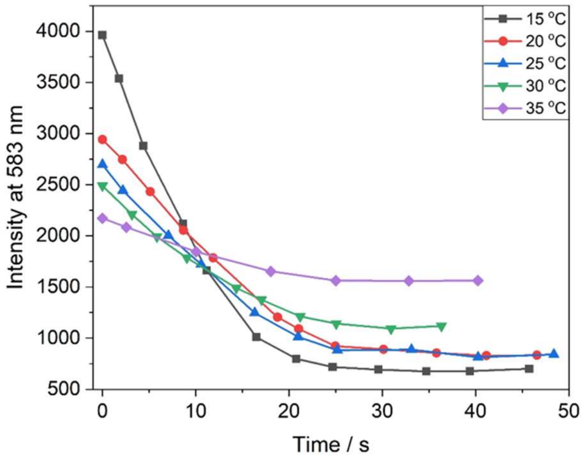

Supplementary Figure 37. Fluorescence intensity at 583 nm of SMEH-CS-SRB in aqueous solution as a function of irradiation (420 nm light, optical power density: 15 mW/cm^2) time at different temperature (15 ºC, 20 ºC, 25 ºC, 30 ºC and 35 ºC, respectively). ([SMEH]$_{initial}$ = 0.15 mM, [CS] = 40 μg/mL, [SRB] = 0.001 mM, λ_{ex} = 560 nm)

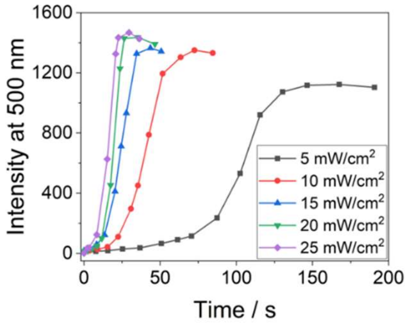

Supplementary Figure 38. Fluorescence intensity at 500 nm of SMEH-CS-C153 in aqueous solution as a function of irradiation time at 15 °C, irradiated by 420 nm light with different power density, 5 mW/cm^2, 10 mW/cm^2, 15 mW/cm^2, 20 mW/cm^2 and 25 mW/cm^2, respectively. ([SMEH]$_{initial}$ = 0.15 mM, [CS] = 40 µg/mL, [C153] = 0.001 mM, λ_{ex} = 425 nm)

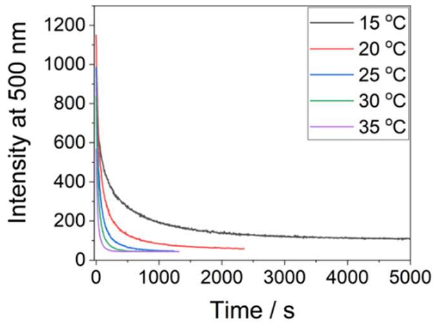

Supplementary Figure 39. Decreasing kinetics of fluorescence intensity at 500 nm of SMEH-CS-C153 after irradiation by 420 nm light (optical power density: 15 mW/cm^2) for 50 s at different temperature (15 °C, 20 °C, 25 °C, 30 °C and 35 °C, respectively) in the dark in aqueous solution. ([SMEH]$_{initial}$ = 0.15 mM, [CS] = 40 µg/mL, [C153] = 0.001 mM, λ_{ex} = 425 nm)

J. Cell cytotoxicity

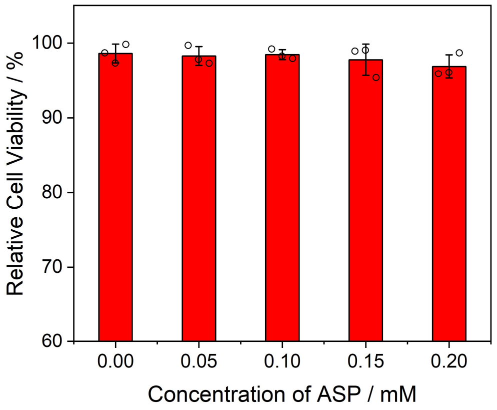

Supplementary Figure 40. Relative cell viabilities of ASP-CS-SRB at different concentrations (concentration ratio among [ASP]:[CS]:[SRB] remain 0.15mM:40µg/mL:0.001mM, concentration in the figure represents [ASP]). n = 3 independent experiments, with the bar data indicating mean ± SD.

K. ^{1}H-NMR, ^{13}C-NMR and HRMS spectra of SMEH

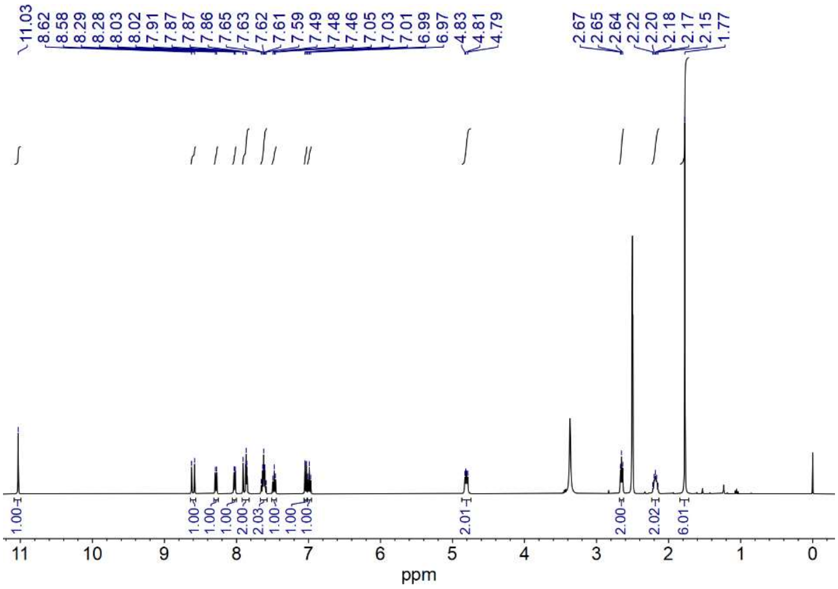

Supplementary Figure 41. ^{1}H-NMR spectra of SMEH (400 MHz, DMSO-d_6).

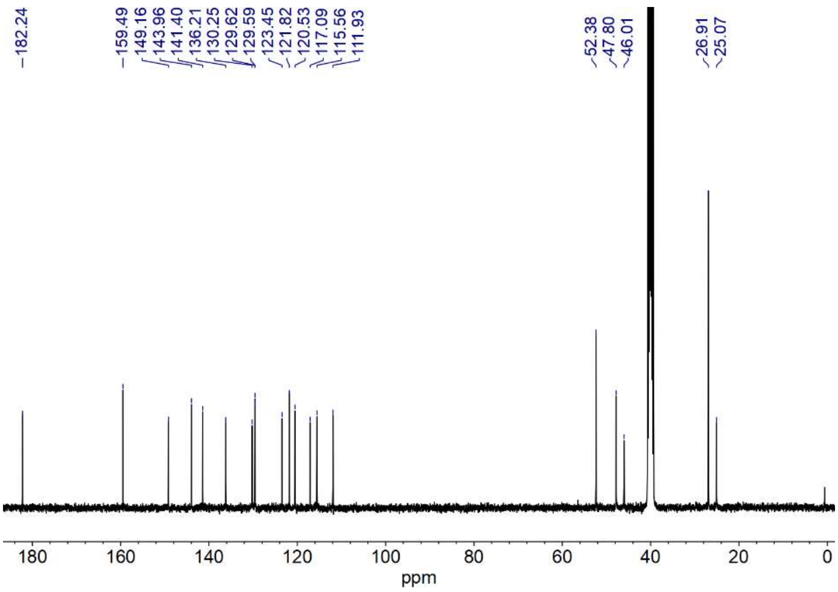

Supplementary Figure 42. ^{13}C-NMR spectra of SMEH (101 MHz, DMSO-d_6).

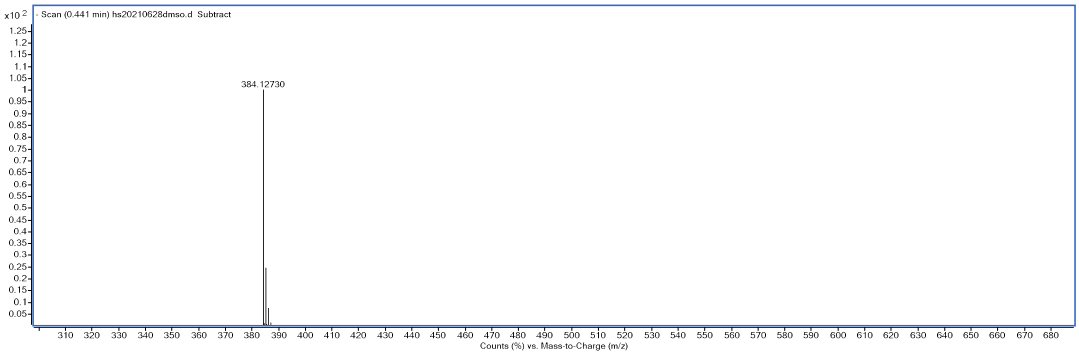

Supplementary Figure 43. HRMS spectra of SMEH.

L. References

1. Shi, Z., Peng, P., Strohecker, D. & Liao, Y. Long-lived photoacid based upon a

photochromic reaction. *J. Am. Chem. Soc.* **133,** 14699−14703 (2011).
2. Maiti, S., Fortunati, I., Ferrante, C., Scrimin, P. & Prins, L. J. Dissipative self-assembly of vesicular nanoreactors. *Nat. Chem.* **8**, 725–731 (2016).
3. Chen, X., Chen, Y., Hou, X., Wu, X., Gu, B. & Liu, Y. Sulfonato-β-cyclodextrin mediated supramolecular nanoparticle for controlled release of berberine. *ACS Appl. Mater. Interfaces* **10**, 24987–24992 (2018).
