# Supplementary material for: Light-fueled transient supramolecular assemblies in water as fluorescence modulators
Source: Nat Commun. 2021 Aug 17;12:4993. doi: 10.1038/s41467-021-25299-8 (PMC8371092; doi:10.1038/s41467-021-25299-8)
Supplement: Supplementary file 3 — Description of Additional Supplementary Files [file 41467_2021_25299_MOESM3_ESM.pdf]

Description of Additional Supplementary Files

File Name: Supplementary Movie 1
Description: The confocal images and fluorescent particle counts of light-induced formation and thermal dissociation of the transient supramolecular assemblies by loading C153.

File Name: Supplementary Movie 2
Description: Dynamic fluorescent variation of loaded TCPE over time during light-induced assembly.

File Name: Supplementary Movie 3
Description: Dynamic fluorescent variation of loaded TCPE over time during thermal dissociation.
